# A Viperin Mutant Bearing the K358R Substitution Lost its Anti-ZIKA Virus Activity

**DOI:** 10.3390/ijms20071574

**Published:** 2019-03-29

**Authors:** Bénédicte Vanwalscappel, Gilles Gadea, Philippe Desprès

**Affiliations:** Université de La Réunion, INSERM U1187, CNRS UMR 9192, IRD UMR 249, Unité Mixte Processus Infectieux en Milieu Insulaire Tropical, Plateforme Technologique CYROI, 97491 Sainte-Clotilde, La Réunion, France; benedicte.vanwalscappel@univ-reunion.fr (B.V.); gilles.gadea@inserm.fr (G.G.)

**Keywords:** innate immunity, ISG, viperin, arbovirus, Zika virus, A549 cells, mutational analysis, protein expression

## Abstract

Interferon-induced viperin (VP) was identified as playing an important role in the innate immune response against Zika virus (ZIKV). The 361 amino acid long human VP protein comprises of a highly conserved C-terminal region, which has been associated with VP antiviral properties against ZIKV. In the present study, we sought to determine whether the very last C-terminal amino-acid residues of VP might play a role in VP-mediated ZIKV inhibition. To address this issue, a recombinant human viperin (rVP^wt^) was overexpressed by transfection in human epithelial A549 cells. We confirmed that transient overexpression of rVP^wt^ prior to ZIKV infection dramatically reduced viral replication in A549 cells. Deletion of the last 17 C-terminal amino acids of VP resulted in a higher expression level of mutant protein compared to wild-type VP. Mutational analysis revealed that residue substitution at positions 356 to 360 with five alanine led to the same phenotype. The charged residues Asp356, Lys358, and Asp360 were then identified to play a role in the weak level of VP^wt^ protein in A549 cells. Mutant VP bearing the D360A substitution partially rescued ZIKV growth in A549 cells. Remarkably, a single Lys-to-Arg substitution at position 358 was sufficient to abrogate VP antiviral activity against ZIKV. In conclusion, our study showed that Asp356, Lys358, and Asp360 may have an influence on biochemical properties of VP. Our major finding was that Lys358 was a key amino-acid in VP antiviral properties against ZIKV.

## 1. Introduction

The recent emergence of mosquito-borne Zika virus (ZIKV) has been associated with an increase in infection severity in humans, thus drawing attention to Zika illness as a public health concern worldwide [1]. ZIKV is an enveloped, single-stranded positive-sense RNA virus that belongs to the flavivirus genus (*Flaviviridae* family) [2]. Other flaviviruses of medical concern are dengue virus (DENV), Japanese encephalitis virus (JEV), yellow fever virus (YFV), West Nile virus (WNV) and Tick-borne encephalitis virus (TBEV) [2,3,4]. ZIKV viral strains are clustered into two major lineages, the African and Asia genotypes [5]. ZIKV replication into the cytosol of the host-cell involves viral RNA translation into a polyprotein precursor that is processed by viral and cellular proteases to produce three structural proteins C, prM, and E and seven non-structural (NS) proteins NS1 to NS5 [6,7]. 

ZIKV infection of human lung epithelial A549 cells with historical strain MR766 (African lineage) and epidemic strain BR15 (Asian lineage) resulted in the activation of a series of interferon-stimulated genes (ISGs) [8]. Viperin (for Virus inhibitory protein endoplasmic reticulum-associated interferon-inducible) has been identified as a major ISG since its expression is induced in response to Type I interferons (IFNs), as well as viral infection [9,10]. VP exerts antiviral activity against a large range of viruses. Several reports have shown the important role of VP in antiviral immunity against flaviviruses including ZIKV [11,12,13,14,15]. However, the mechanisms by which VP restricts viral infection differ depending on the pathogen [16,17,18,19,20,21]. In an effort to better understand the molecular basis of VP antiviral action against flaviviruses, it has been observed that VP colocalizes with viral replication complexes, which regroup the NS proteins such as serine protease-NTPase/helicase NS3 [20,21]. Panayiotou et al. demonstrated that VP could interact with the NS3 proteins from JEV, TBEV, YFV, and ZIKV and that the VP-mediated degradation of NS3 might account for the restriction of ZIKV replication [13]. The VP-mediated ZIKV inhibition could also be attributable to a lower production of viral RNA molecules. Indeed, VP can catalyze the conversion of cytidine triphosphate (CTP) into 3′-deoxy-3′,4′-didehydro-CTP (ddhCTP) that acts as a chain terminator for the RNA-dependent RNA polymerase NS5, thus leading to an arrest in viral RNA replication [22].

VP, also known as RSAD2 (for radical SAM domain-containing 2), is a conserved protein with a predicted molecular mass of 42 kDa [10]. The 361 amino acid long VP protein comprises three distinct domains: A variable amino-terminal domain with an amphipathic α-helix and a leucine zipper domain, a conserved central domain containing a radical S-adenosyl methionine (SAM) domain and a highly conserved C-terminal domain [9,10]. The role of the radical SAM domain in VP antiviral function remains somewhat elusive. 

It has been reported that the deletion of the C-terminus amino-acid residue W361 of human viperin abrogates its antiviral activity against hepatitis C virus belonging to the *Flaviviridae* family [23]. Furthermore, recent works have demonstrated that VP protein mutants lacking the last C-terminal amino-acid residues are attenuated in their ability to inhibit flavivirus infection [12,14,20]. In the present study, we investigated the role of amino-acid residues 356 to 360 in VP antiviral properties against ZIKV in human epithelial A549 cells. We showed that the three amino acids at positions 356, 358, and 360 may play an important role in biochemical and antiviral properties of VP.

## 2. Results and Discussion

### 2.1. The Last C-Terminal Residues at Positions 356, 358, and 360 Influence VP Protein Level

**Figure 1:**
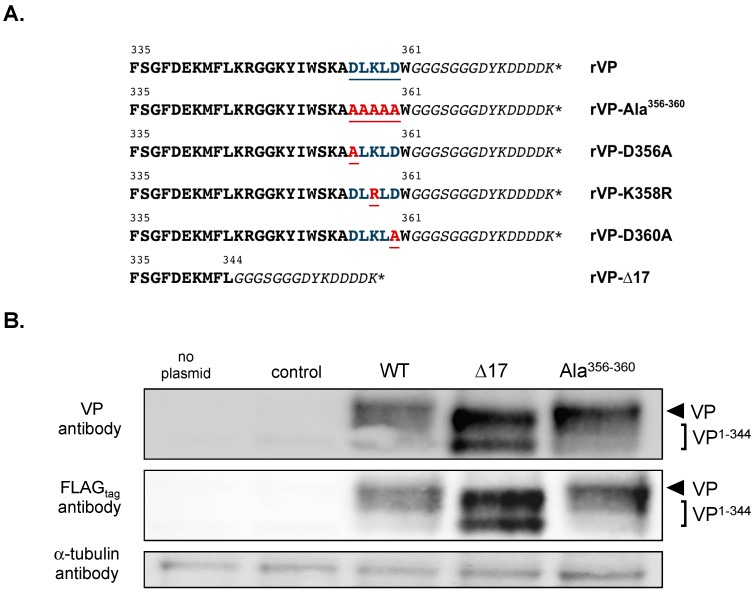
Analysis of rVP mutant expression in A549 cells. In (**A**), sequence alignment of the C-terminal part (VP-335 to VP-361) of recombinant human viperin (rVP^wt^) and its mutants used in this study is shown. The five amino acid residues 356 to 360 mutagenized are highlighted in blue and underlined. The substitutions are highlighted in red and underlined. The glycine-serine spacer and the FLAG_tag_ sequence adjacent to the C-terminus of rVP (W-361) are shown in italics. In (**B**), the analysis of rVP expression by immunoblot assay is shown. A549 cells were transfected at 18 h with pcDNA3/rVP (WT), pcDNA3/rVP-∆17 (VP^1–344^) or pcDNA3/rVP-Ala^356–360^ (Ala^356–360^). As controls, cells were transfected with pcDNA3/RenLUC (control) or mock-transfected (no plasmid). The α-tubulin protein served as a protein loading control.

It has been reported that the C-terminal amino-acid residues of VP might play a major role in its antiviral action against flaviviruses [12,14,20]. To address the role of the five residues located at positions 356 to 360 in VP-mediated ZIKV inhibition, we used a vector plasmid pcDNA3 which overexpressed rVP^wt^ tagged with a C-terminal FLAG epitope (Figure 1A). It has recently been reported that viral clone BR15 derived from a Brazilian epidemic ZIKV strain BeH819015 replicated efficiently in human epithelial A549 cells [8]. Here, we show that IFN-β stimulates expression of endogenous VP mRNA indicating that A549 epithelial cells have the ability to produce VP (Figure S1). rVP^wt^ anti-ZIKV activity was then validated in human epithelial A549 cells transfected at 18 h with pcDNA3/rVP and then infected 24 h with ZIKV strain BR15 (Figure S2). Analysis of viral growth showed that rVP^wt^ transient expression prevents ZIKV infection in agreement with a previous report [14].

We investigated the role of the last C-terminal amino-acid residues in VP antiviral action against ZIKV. Mutations D356A, L357A, K358R, L359A, and D360A were introduced into the rVP^wt^ sequence of pcDNA3/rVP in order to generate a mutant pcDNA3/rVP-Ala^356–360^ (Figure 1A). Given that VP requires its 17 amino-acid C-terminal residues for its anti-DENV activity [20], we also engineered a mutant plasmid pcDNA3/rVP-∆17 as a control (Figure 1A). We first analyzed the expression of the two mutants rVP with wild-type rVP in A549 cells transfected 18 h with either pcDNA3/rVP-∆17, pcDNA3/rVP-Ala^356–360^, or pcDNA3/rVP. Immunoblot assay was performed on cell lysates using anti-FLAG_tag_ or anti-VP antibody (Figure 1B). The use of an antibody against an N-terminal epitope of VP (anti-VP) or its C-terminal tag gave a comparable pattern of protein expression. We observed that rVP-∆17 mutant migrated faster than rVP^wt^ and rVP-Ala^356–360^ due to the deletion of the last C-terminal amino acids. A smaller form of rVP-∆17 was also detected in cell lysates but its origin remains to be understood.

**Figure 2:**
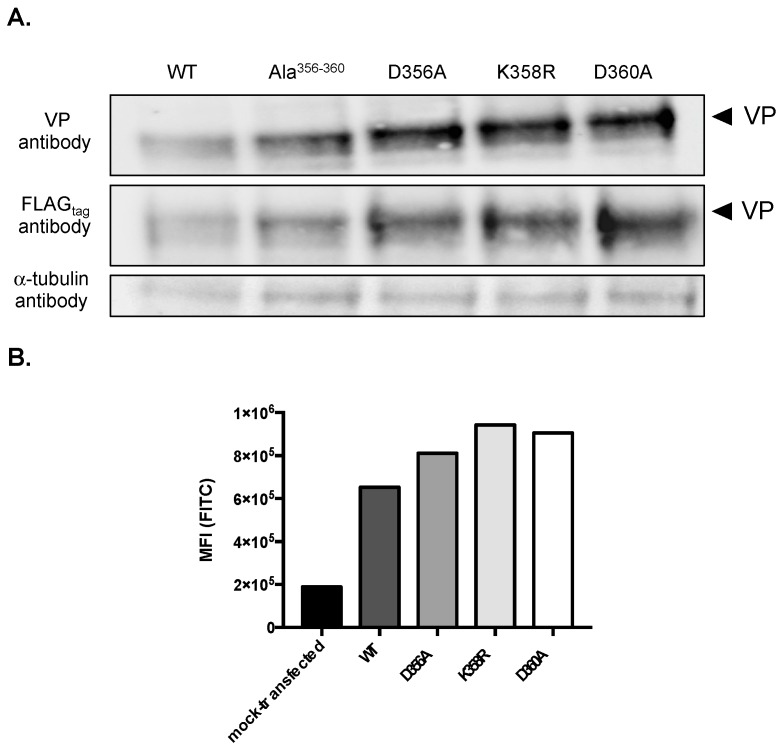
Expression of mutant rVPs bearing the D356A, K358R, and D360A substitutions in A549 cells. A549 cells were transfected at 18 h with pcDNA3/rVP (WT), pcDNA3/rVP-Ala^356–360^ (Ala^356–360^), pcDNA3/rVP-D356A (D356A), pcDNA3/rVP-K358R (K358R), or pcDNA3/rVP-D360A (D360A). In (**A**), an immunoblot assay was performed with anti-FLAG_tag_ or anti-VP antibody. The α-tubulin protein served as a protein loading control. In (**B**), FACS analysis using anti-FLAG_tag_ antibody was performed on mock-transfected (control) or transfected cells and the mean of fluorescence intensity (MFI) of rVP-positive cells is shown.

We observed higher protein levels for rVP-Ala^356–360^ and rVP-∆17 in A549 cells than for rVP^wt^ (Figure 1B). As residues Asp356, Leu357, Lys358, Leu359, and Asp360 might play a role in VP expression, we decided to generate mutant plasmids pcDNA3/rVP bearing the individual D356A, K358R, or D360A substitution (Figure 1A). The expression of the three mutant rVPs was assessed in A549 cells by immunoblot assay and FACS analysis (Figure 2). Both rVP^wt^ and rVP-Ala^356–360^ were used as controls. The immunoblot assay using anti-FLAG_tag_ or anti-VP antibody, revealed that protein levels of rVP-D356A, rVP-K358R, and rVP-D360A were increased compared to VP^wt^ (Figure 2A). FACS analysis confirmed the higher protein levels of mutant rVPs (Figure 2B). These results suggest that each of the charged residues Asp356, Lys358, and Asp360 may have an influence on the VP protein level in A549 cells.

**Figure 3:**
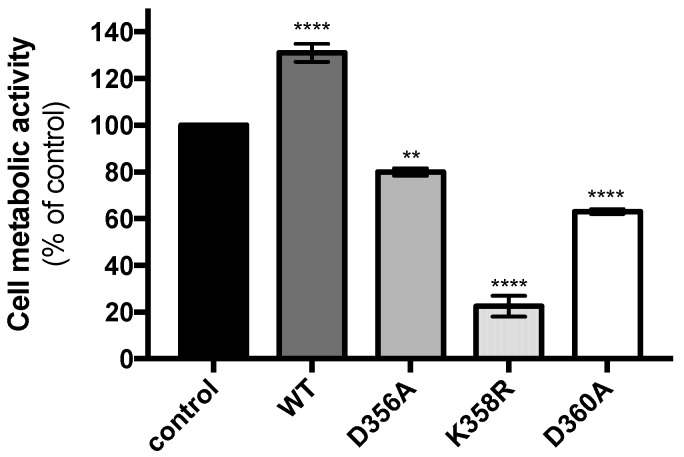
Metabolic activity of cells expressing rVP mutants. A549 cells were transfected at 18 h with pcDNA3/rVP (WT), pcDNA3/rVP-D356A (D356A), pcDNA3/rVP-K358R (K358R), or pcDNA3/rVP-D360A (D360A) and cell metabolic activity was measured 24 h later (42 h post-transfection) using a MTT assay. As a plasmid control, cells were transfected with pcDNA3/*RenLUC* (control). Viability was expressed as the percentage of cell metabolic activity in assay relative to that in control. Results were expressed as the mean (± SEM) from three independent experiments.

We next sought to determine whether the expression of mutant rVPs may have an effect on cell metabolic activity. A549 cells were transfected 18 h with plasmids expressing rVP^wt^ or its mutants and cell metabolic activity was measured 24 h later by a MTT assay (Figure 3). Expression of the rVP-D356A and rVP-D360A resulted in a weak or moderate reduction in cell metabolic activity whereas rVP^wt^ showed no effect. Interestingly, mutant rVP bearing the K358R substitution was responsible for a severe decrease in cell metabolic activity. Thus, the expression of rVP-K358R has a greater impact on the metabolism of A549 cells.

**Figure 4:**
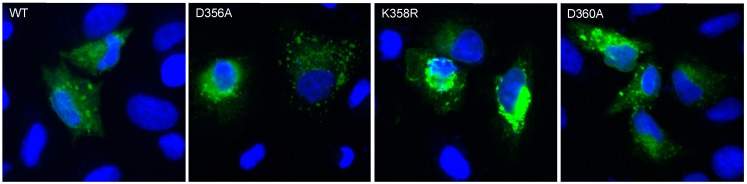
Intracellular expression of VP mutants. A549 cells were transfected at 18 h with plasmids expressing rVP^wt^, rVP-D356A, rVP-K358R, or rVP-D360A. Permeabilized cells were stained with anti-FLAG_tag_ antibody (green) as the primary antibody. The nuclei were stained with DAPI (Blue). Immunostained cells were visualized with a fluorescent microscope. The same magnification of ×200 was used throughout.

We asked whether the effect of rVP-K358R on cell metabolic activity relates to a change in its intracellular distribution in A549 cells. It has been reported that VP localizes to the cytosolic face of the ER compartment [24]. We visualized the expression of rVP^wt^ and its mutants at positions 356, 358, and 360 in A549 cells by immunofluorescence (IF) analysis using anti-FLAG_tag_ antibody (Figure 4). A similar pattern had been observed between rVP-D356A, rVP-D360A and rVP^wt^. For the rVP-K358R mutant, a pronounced perinuclear fluorescent signal was visualized suggesting that the Lys-to-Arg substitution at position 358 might have had an influence on the subcellular distribution of rVP in A549 cells.

### 2.2. The Residues K358 and D360 Influence VP-Mediated ZIKV Inhibition

We investigated whether the substitutions at positions 356, 358, and 360 may have had an influence on VP antiviral properties. To first determine whether expression of mutant rVPs had an effect on cell viability at the time-point of ZIKV infection, caspase-3/7 activity was measured on cell lysates at 18 h post-transfection using a caspase 3/7 kit assay (Figure S3). We observed that the expression of the rVP-D356A, rVP-K358R, and rVP-D360A resulted in a weak increase in caspase-3 activity compared to rVP^wt^. Thus, the three mutant rVPs were suitable for a comparative analysis of their antiviral activity against ZIKV.

**Figure 5:**
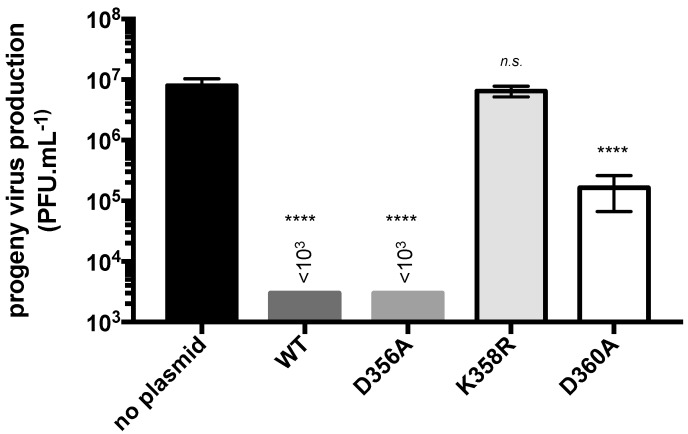
Antiviral activity of rVP mutants against ZIKV. A549 cells were transfected at 18 h with plasmids expressing rVP^wt^ (WT), rVP-D356A (D356A), rVP-K358R (K358R), or rVP-D360A (D360A) or mock-transfected (no plasmid). Cells were infected at 24 h with ZIKV strain BR15 at MOI of 1. Virus progeny production was determined by a plaque forming assay on Vero cells. Results were expressed as the mean (± SEM) of three independent assays. *p*-values were determined on the comparison with no plasmid. **** *p* < 0.0001, n.s, not significant, *p* < 0.05.

To evaluate the antiviral effect of the mutant rVPs against ZIKV, A549 cells were transfected at 18 h with plasmids expressing either rVP-D356A, rVP-K358R, or rVP-D360A and then infected at 24 h with the ZIKV strain BR15 (Figure 5). Transfection with pcDNA3/rVP^wt^ served as a positive control. As expected, the expression of rVP^wt^ prior to ZIKV infection reduced virus progeny production by at least 4 log. A comparable ZIKV inhibition was observed with rVP-D356A. In contrast, rVP-D360A was much less efficient at inhibiting ZIKV with a virus progeny production, which reduced by only 2 log. Unexpectedly, rVP-K358R showed no antiviral activity against ZIKV suggesting a critical role for residue Lys358 in VP-mediated ZIKV inhibition. Because rVP-K358R expression can greatly affect the metabolic activity of A549 cells, we tested whether rVP-K358R affects viability of ZIKV-infected cells. Using a caspase 3/7 kit assay, we only detected a weak increase in the rate of cell death in ZIKV-infected A549 cells expressing rVP-K358R compared to rVP^wt^ (Figure S4). This suggested that the lack of anti-ZIKV activity was not related to increased apoptosis of A549 cells expressing rVP-K358R. Taken together, these results showed that both Lys358 and Asp360 influenced VP antiviral properties. We found that a single Lys-to-Arg substitution at position 358 resulted in a complete lack of VP antiviral activity against ZIKV. 

**Figure 6:**
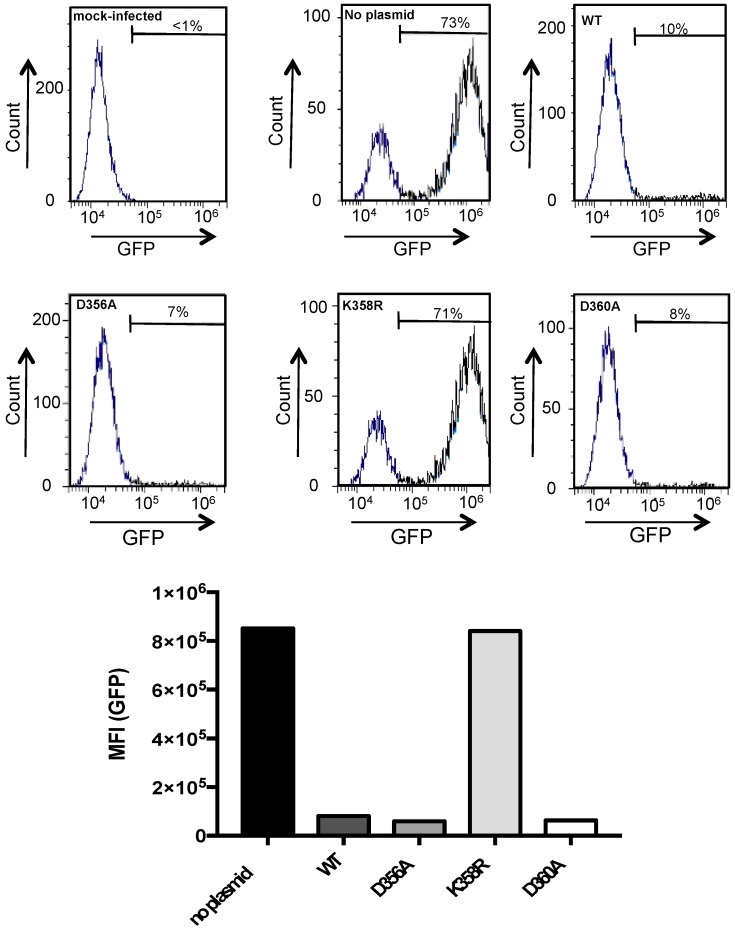
Antiviral action of mutant rVPs against ZIKV replication. A549 cells were transfected 18 h with plasmids expressing rVP^wt^, rVP-D356A, rVP-K358R, or rVP-D360A or mock-transfected (no plasmid). Cells were infected 24 h with ZIKV_GFP_ at MOI of 2. The percentage of GFP-positive cells and the MFI were determined by FACS analysis.

We asked whether the lack of anti-ZIKV activity of rVP-K358R relates to its inability to inhibit viral replication. To investigate this issue, we used the recombinant GFP-expressing ZIKV (ZIKV_GFP_) derived from ZIKV historical African strain MR766 [14,25,26]. We previously demonstrated that GFP is a reliable reporter protein that monitors viral replication inside the infected host-cells [14,25,26]. A549 cells were transfected at 18 h with plasmids expressing rVP^wt^ or mutant rVPs and then infected 24 h with ZIKV_GFP_ at MOI (multiplicity of infection) of 2 (Figure 6). FACS analysis, showed that the expression of rVP^wt^ and its two mutant rVP-D356A and rVP-D360A reduced the rate of GFP-positive A549 cells by 90% compared to mock-transfected cells. In contrast, the expression of rVP-K358R showed no effect on ZIKV replication efficiency in A549 cells. Together, these results showed that a Lys-to-Arg substitution at position 358 abrogated VP ability to inhibit ZIKV replication regardless of viral strain origin.

### 2.3. Concluding Remarks

Viperin has been evaluated as one of the ISG-encoded proteins playing a major role in the antiviral innate immune response against ZIKV [12,13,14]. The underlying molecular mechanisms of viperin antiviral action against ZIKV have been partially resolved. Viperin was found to bind to and cause degradation of viral NS3 protein by a proteasome-dependent proteolytic pathway [13]. It has also been found that viperin is able to produce a chain terminator for the RNA-dependent RNA polymerase NS5 [22]. Although a direct connection between these two antiviral mechanisms is not obvious, it is likely that viperin inhibits ZIKV by interrupting viral RNA synthesis inside the host-cell. 

The purpose of our study was to investigate the role of the amino-acid residues 356 to 360 in viperin antiviral properties against ZIKV. The viperin antiviral properties were analyzed in human epithelial A549 cells, which were transient transfected with a plasmid vector overexpressing recombinant human viperin. We confirmed that the expression of our recombinant viperin greatly inhibited ZIKV growth in A549 cells. Immunoblot analysis detected a weak level of recombinant viperin protein in transiently transfected A549 cells. It has been reported that viperin could be subjected to proteolysis by the proteasome [11]. Mutational analysis revealed that the C-terminal amino-acid residues 356 to 360 may have had an influence on the viperin protein level. To our knowledge, this is the first report suggesting a role for the last C-terminal amino-acid residues in the biochemical properties of viperin. Given that substitutions D356A, K358R, and D360A increased the level of viperin expression in A549 cells, the charged amino-acid residues at positions 356, 358, and 360 might have played an important role in viperin stability. Understanding the mechanisms by which Asp356, Lys358, and Asp360 influence the stability of human viperin is a critical issue that will be the subject of further investigation.

An important observation was that a mutant viperin bearing the K358R substitution lacked anti-ZIKV activity whereas D360A substitution only resulted in a partial loss of its antiviral action. Our data identified Lys358 as a key amino acid for viperin antiviral action against ZIKV. Mechanisms by which the C-terminal amino acid residue Lys358 influences viperin antiviral properties remain to be understood. It has been reported that the ER membrane-associated viperin has ability to self-assemble leading to formation of oligomers [24]. Furthermore, it was extrapolated that protein oligomerization links to the C-terminal domain of viperin [24]. Future studies will investigate whether the lack of anti-ZIKV activity of a mutant viperin bearing the K358R substitution could be related to a defect of protein oligomerization. It is also of interest to determine whether viperin could be subjected to a post-translational modification though a protein-protein interaction-dependent mechanism. Interestingly, sequence analysis of the last C-terminal amino-acid residues of human viperin identified the tetrapeptide Leu357-Lys358-Leu359-Asp360 as a possible SUMO-interaction motif ψKxD where ψ represented a hydrophobic residue. SUMOylation is a post-translational modification of proteins in response to internal and external stimuli including viral infection [27,28,29,30,31]. SUMOylation results in the formation of an isopeptide bond between the C-terminal SUMO di-glycine motif and the ξ-amino group of a lysine residue within the SUMO motif of the target protein [32,33]. 

It is tempting to infer that the tetrapeptide Leu357-Lys358-Leu359-Asp360 at the C-terminal region of viperin was subjected to a post-translational modification by SUMOylation. The involvement of Asp360 in a putative SUMO-interaction motif LKLD is based on the fact that a mutant viperin bearing a D360A substitution was able to partially rescue ZIKV growth in A549 cells. The engagement of Lys358 into a mechanism of SUMOylation is supported by the finding that the Lys-to-Arg change which retained a positive charge at position 358 abrogated the ability of VP to inhibit ZIKV. We noted that the expression of mutant viperin bearing the K358R substitution resulted in a marked reduction in A549 cell metabolic activity whereas wild-type viperin showed no effect on cell metabolic activity. The metabolic activity reduction was associated with a particular profile in the cellular distribution of such a mutant viperin. Whether there is a potential link between the lack of anti-ZIKV activity of VP-K358R and its effect on cell metabolism is still an open issue. Further studies will determine whether Lys358 is a key amino acid in both biochemical and antiviral properties of viperin.

## 3. Materials and Methods 

### 3.1. Cell Lines and Virus

Vero cells (ATCC, CCL81) were cultured in Minimum Essential Media (MEM) supplemented with 5% heat-inactivated fetal bovine serum (FBS). A549-Dual^TM^ cells (Invivogen Inc, Toulouse, France) designated hereafter as A549 cells were grown in MEM medium supplemented with 10% heat-inactivated FBS, non-essential amino acids, 10 µg mL^−1^ blasticidin and 100 mg mL^−1^ zeocin (InvivoGen, Toulouse, France). The molecular clone BR15 derived from epidemic Brazilian ZIKV strain BeH819015 and the recombinant GFP-expressing ZIKV_GFP_ derived from historical African ZIKV strain MR766-NIID have been described elsewhere [8,25]. Virus stocks were grown on Vero cells. Infectivity of ZIKV was determined by a plaque-forming assay on Vero cells [34]. Cells were routinely infected with ZIKV at the MOI of 1 or 2 plaque-forming units (PFU) per cell.

### 3.2. Antibodies

Mouse anti-viperin antibody clone MaP.VIP was purchased from Sigma-Aldrich (Saint-Quentin-Fallavier, France). The mouse anti-DDDDK tag mAb (anti-FLAG_tag_ antibody), goat anti-mouse and goat anti-rabbit immunoglobulin-horseradish peroxidase (HRP) conjugated secondary antibodies were purchased from Abcam (Cambridge, UK). DAPI was purchased from Euromedex (Souffelweyersheim, France). The rabbit anti-α tubulin polyclonal Ab was purchased from Santa Cruz Biotechnology (Dallas, Texas, USA).

### 3.3. Vector Plasmids Expressing Viperin and Its Mutants

The coding sequences for human viperin (Genbank accession number AAL50053) or its mutants followed in frame by a glycine-serine spacer and the FLAG_tag_ sequence (Figure 1) were synthesized and cloned into Nhe I and Not I restriction sites of the pcDNA 3.1 (+) Neo plasmid by Genecust (Luxembourg). The recombinant plasmids were verified by sequencing (Genecust, Luxembourg). Plasmids were transfected in A549 cells using Lipofectamin^TM^ 3000 according to the manufacturer’s instructions (Thermo Fisher Scientific, Illkirch-Grafenstaden, France).

### 3.4. Flow Cytometry Analysis 

A549 cells (1.5 × 10^5^) were fixed with 3.7% paraformaldehyde (PFA) in phosphate buffered saline (PBS) for 10 min. For the detection of rVPs, cells were permeabilized with 0.15% of Triton X-100 in PBS and then incubated with anti-FLAGtag antibody (dilution 1:2000) for 1 h at room temperature. Goat anti-mouse Alexa Fluor 488 IgG antibody was used as a secondary antibody. For ZIKV_GFP_, infected cells were fixed and then observed for GFP expression. For each assay, 10^4^ cells were analyzed by flow cytometry (CytoFLEX, Beckman Coulter, Brea, California, USA) using FlowJo software (BD Bioscience, Le Pont-de-Claix, France).

### 3.5. Immunofluorescence Assay

A549 cells (1.5 × 10^5^) were grown on coverslips and fixed with 3.7% PFA for 10 min and permeabilized with 0.15% Triton X-100 in PBS for 4 min. Cells were stained using the mouse anti-FLAG_tag_ antibody and followed by goat anti-mouse Alexa Fluor 488 IgG secondary antibody. The nucleus was stained with DAPI and the cells were visualized with a Nikon Eclipse E2000-U microscope (Nikon, Lisses, France). Images were captured and treated using a Hamamatsu ORCA-ER camera (Hamamatsu, Japan) and the imaging software NIS-Element AR (Nikon, Lisses, France).

### 3.6. Immunoblot Analysis

Cells were lysed in urea buffer and cell lysates were separated by SDS-PAGE, transferred to nitrocellulose membranes and probed with primary antibody. The membranes were washed and then probed with goat anti-mouse or anti-rabbit immunoglobulin-HRP conjugated second antibody. Blots were revealed with ECL detection reagents.

### 3.7. MTT Assay

A549 cells were cultured in a 96-well plates at a density of 2 × 10^4^ cells per well. Cell monolayers were rinsed with PBS 1× and incubated with culture growth medium mixed with 5 mg·mL^−1^ MTT (3-[4,5-dimethylthiazol-2-yl]-2,5-diphenyltetrazolium bromide) solution for 1 h at 37 °C. MTT medium was removed and the formazan crystals were solubilized with dimethyl sulfoxide (DMSO). Absorbance was measured at 570 nm with a background subtraction at 690 nm. 

### 3.8. Caspase 3/7 Activity

A549 cells were cultured in a 96-well plates at a density of 2 × 10^4^ per well. Caspase 3/7 activity in raw cell lysates was measured using a Caspase Glo® 3/7 assay kit (Promega, Charbonnières-les-bains, France) according to the manufacturer’s protocol. Caspase activity was quantified by luminescence using a FLUOstar Omega Microplate Reader (BMG LABTECH, Champigny-sur-Marne, France).

### 3.9. Statistical Analysis

Values of independent experiments were analyzed by ANOVA or t-tests as appropriate. Values of *p* < 0.05 were considered statistically significant. Asterisks indicate that the differences were statistically significant: **** *p* < 0.0001, *** *p* < 0.001, ** *p* < 0.01, *p < 0.1.

## Supplementary Materials

Supplementary materials can be found at https://www.mdpi.com/1422-0067/20/7/1574/s1.

## Abbreviations

CTP	Cytidine triphosphate
ddhCTP	3′-deoxy-3′, 4′ didehydro-CTP
DENV	Dengue virus
DMSO	Dimethyl sulfoxide
ER	Endoplasmic reticulum
FBS	Fetal bovine serum
HCV	Hepatitis C virus
HRP	Horseradish-peroxidase
IFNs	Type I interferons
ISG	Interferon-stimulated gene
IF	Immunofluorescence
IFN	Type I interferons
IU	International unit
JEV	Japanese encephalitis virus
kDa	Kilo Dalton
mAb	Monoclonal antibody
MEM	Minimum Essential Media
MFI	Mean fluorescence intensity
MOI	Multiplicity of infection
MTT	3′-[4,5-dimethylthiazol-2-yl]-2,5-diphenyltetrazolium bromide
NS	Non-structural
PBS	Phosphate buffered saline
PFA	Paraformaldehyde
RSAD2	Radical SAM domain-containing 2
rVP^wt^	Recombinant human viperin
SAM	S-adenosyl methionine
SDS-PAGE	Sodium dodecyl sulfate poly-acrylamide gel electrophoresis
TBEV	Tick-borne encephalitis virus
VP	Viperin
WNV	West Nile virus
YFV	Yellow fever virus
ZIKV	Zika virus
