# Supplementary material for: A Viperin Mutant Bearing the K358R Substitution Lost its Anti-ZIKA Virus Activity
Source: Int J Mol Sci. 2019 Mar 29;20(7):1574. doi: 10.3390/ijms20071574 (PMC6480927; doi:10.3390/ijms20071574)
Supplement: Supplementary file 1 [file ijms-20-01574-s001.pdf]

Supplementary Materials:

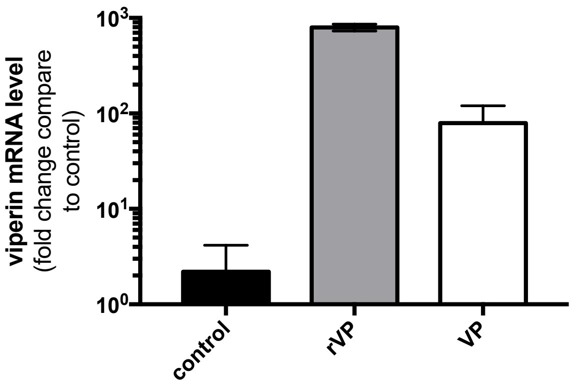

Figure S1. Detection of VP mRNA transcripts by RT-qPCR. Total RNA was extracted A549 cells incubated 18 h with IFN-β (10,000 IU.mL^{-1}) (VP) or mock-treated (control) using RNeasy kit (Qiagen). As a positive control, cells were transfected 18 h with pcDNA3/rVP (rVP). RNA was transcribed into cDNA with a random primer p(dN)$_6$. VP mRNA was quantified by RT-qPCR using a couple of primers targeting the 5′-end region of VP mRNA as described elsewhere [1]. The data are normalized to GAPDH mRNA and expressed as a fold-change relative to control.

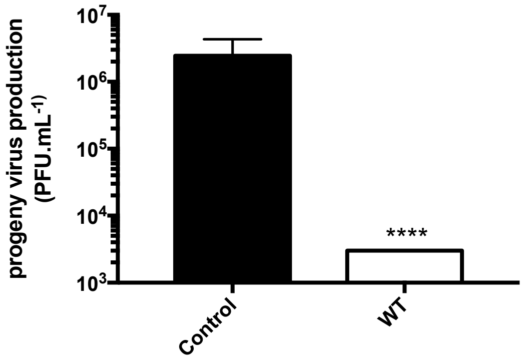

Figure S2. Antiviral action of rVP^{wt} against epidemic strain BR15 of ZIKV. A549 cells were transfected 18 h with pcDNA3/rVP or mock-transfected (control), and then infected with ZIKV strain BR15 at MOI of 1. At 24 h p.i., virus progeny production was determined by plaque forming assay on Vero cells. The results are the mean (± SEM) of two independent assays. *p*-values were determined on the comparison with mock-transfected cells (*t*-test).

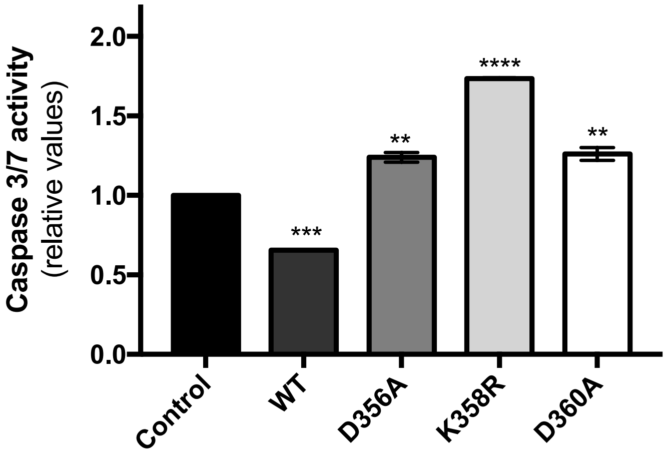

Figure S3. Caspase 3 activity in A549 cells expressing rVPs. A549 cells were transfected 18 h with pcDNA3/rVP (WT), pcDNA3/rVP-D356A (D356A), pcDNA3/rVP-K358R (K358R), or pcDNA3/rVP-D360A (D360A). As a negative control, cells were transfected with pcDNA3/*Ren*LUC (control). The caspase 3/7 activity in cell extracts was determined at 18 h post-transfection. Results are shown as a fold-change relative to control. *p*-values were determined on the comparison with control (Anova). Values are given as the mean of triplicates.

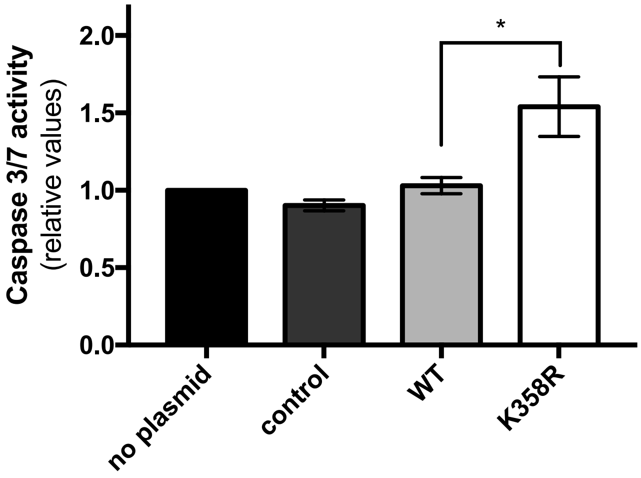

Figure S4. Caspase 3 activity in A549 cells expressing rVPs and infected with ZIKV. A549 cells were transfected 18 h with pcDNA3/rVP (WT), pcDNA3/rVP-K358R (K358R) or pcDNA3/*Ren*LUC (control) and then infected with ZIKV at MOI of 1. Caspase 3/7 activity in cell extracts was determined at 24 h later (42 h post-transfection). Results are shown as a fold-change relative to no plasmid. *p*-value was determined on the comparison with rVP wild-type (Anova). Values are given as the mean of triplicates.

References

1. Muramatsu, D.; Kawata, K.; Aoki, S.; Uchiyama, H.; Okabe, M.; Miyazaki, T.; Kida, H.; Iwai, A. Stimulation with the Aureobasidium pullulans-produced beta-glucan effectively induces interferon stimulated genes in macrophage-like cell lines. *Sci. Rep.* **2014**, *4*, 4777.
